# Tumor Angiogenesis and Vascular Patterning: A Mathematical Model

**DOI:** 10.1371/journal.pone.0019989

**Published:** 2011-05-27

**Authors:** Rui D. M. Travasso, Eugenia Corvera Poiré, Mario Castro, Juan Carlos Rodrguez-Manzaneque, A. Hernández-Machado

**Affiliations:** 1 Centro de Física Computacional, Departamento de Física, Universidade de Coimbra, Coimbra, Portugal; 2 Departamento de Física y Química Teórica, Facultad de Química, Universidad Nacional Autónoma de México, México; 3 GISC and Grupo de Dinámica No-lineal (DNL), Escuela Téc. Sup. de Ingeniería (ICAI), Universidad Pontificia Comillas, Madrid, Spain; 4 GENYO (Pfizer-University of Granada-Andalusian Government Centre for Genomics and Oncological Research), Granada, Spain; 5 Departament ECM, Facultat de Fsica, Universitat de Barcelona, Barcelona, Spain; University of South Florida College of Medicine, United States of America

## Abstract

Understanding tumor induced angiogenesis is a challenging problem with important consequences for diagnosis and treatment of cancer. Recently, strong evidences suggest the dual role of endothelial cells on the migrating tips and on the proliferating body of blood vessels, in consonance with further events behind lumen formation and vascular patterning. In this paper we present a multi-scale phase-field model that combines the benefits of continuum physics description and the capability of tracking individual cells. The model allows us to discuss the role of the endothelial cells' chemotactic response and proliferation rate as key factors that tailor the neovascular network. Importantly, we also test the predictions of our theoretical model against relevant experimental approaches in mice that displayed distinctive vascular patterns. The model reproduces the in vivo patterns of newly formed vascular networks, providing quantitative and qualitative results for branch density and vessel diameter on the order of the ones measured experimentally in mouse retinas. Our results highlight the ability of mathematical models to suggest relevant hypotheses with respect to the role of different parameters in this process, hence underlining the necessary collaboration between mathematical modeling, in vivo imaging and molecular biology techniques to improve current diagnostic and therapeutic tools.

## Introduction

Sprouting angiogenesis - the process by which new blood vessels grow from existing ones - is a ubiquitous phenomenon in health and disease of higher organisms [1]. It plays a pivotal role in diverse processes from embryo development to wound healing, from diabetic retinopathy to tumor growth [2]. Regarding tumor angiogenesis, the idea that the successful establishment of a solid tumor depends on neovascularization has given rise to many cancer therapies conceived to inhibit the tumor vasculature in order to deprive it from oxygen and nutrients [1], [3]–[5]. Evidence has shown that, when applied alone, anti-angiogenic factors have not given the expected results [6]. On the other hand, when anti-angiogenic factors are applied in combination with cytotoxic therapies (chemotherapy and radiation), they have proven to reinforce the efficiency of therapies and to produce an increase in survival [7]. Recently, the concept of normalizationof tumor vasculature has been proposed [8]. It is suggested that certain anti-angiogenic agents can transiently normalize the abnormal tumor vasculature, to make more efficient he delivery of drugs (provided by chemotherapy) and the delivery of oxygen (that enhances the efficiency of radiation therapy). A measurement for vascular normalization has also been proposed [9].

Recent progresses on high resolution microscopy [10] have given the hope that cancer progression can be monitored, at least regarding the characteristics of its associated vasculature. The concurrence of sophisticated image-tracking systems and advanced mathematical models, would provide a relevant tool to perform early prediction and diagnosis, and could help to define the proper therapies to be followed.

Mathematical models of tumor induced angiogenesis have been fundamentally of two types: either microscopic descriptions accounting for cell dynamics [11]–[14], or coarse grained descriptions based on diffusion equations [14]–[16]. The study of the emergent behavior of a newly formed vascular network needs a unified approach, as recently noticed in literature [18]. Hence, the final morphology of the network depends on both phenomenawhich occur at the cell level (*e.g.* the activation and subsequent sprouting of new branches) and on the large scale collective movements of the cells due to endothelial cell proliferation and the tissue properties [19], [20]. We present a multi-scale phase-field model that combines the benefits of a continuum physics description and the capability of tracking individual tip cells. The qualitative and quantitative results of the model are compared with experimental results reported in literature.

### Biological description

The process of tumor angiogenesis starts when endothelial cells of existing capillaries acquire the tip cell phenotype by the action of a protein cocktail produced by tumor and related-tissue cells, generally induced by a hypoxic microenvironment. Tip cells lead the growth of new capillaries in conjunction with further endothelial cells which acquire the stalk cell phenotype. The migration of endothelial tip cells is directed towards increasing concentrations of relevant growth factors. These, such as VEGF (Vascular Endothelial Growth Factor), have a three-fold role at the cell scale: (i) to trigger the permeability of the capillaries and the subsequent activation of the tip cell phenotype, (ii) to promote migration of tip cells in the direction of its gradient, and (iii) to promote the proliferation and survival of the stalk endothelial cells. The angiogenic role of VEGF is opposed by different anti-angiogenic factors in the tissue.

Tip migration is associated with the production of extracellular MMPs (Matrix MetalloProteinases) that are responsible for the remodeling of nearby ECM (ExtraCellular Matrix), and that affects the affinity of VEGF species towards different extracellular locations. This has relevant consequences in the bio-availability of VEGF [17].

The different isoforms of VEGF are distinguishable by the degree in which they anchor to negativelly-charged molecules in the ECM or the cell surface [21], and the action of MMPs appears relevant for the balance of these VEGF species. The presence of isoforms with different abilities for diffusing in the ECM is essential for the creation of a functional capillary network [22] and for the determination of specific vascular patterns [23].

After an opened path in the ECM is generated by the tip cells, a reorganization of the stalk proliferating cells is required in order to form a new lumen for blood circulation, in precise coordination with pericytes and other stromal components. Further processes such as anastomosis (connection between different branches on the network), the action of pressure forces and the intrinsic mechanical properties of the tissue, contribute to the formation of the new vessel network and are finely tuned to determine vascular patterning [24].

## Methods

### Description of the mathematical model

In this work the dynamics of the interface between the newly formed capillaries and the stroma is treated with a phase-field model formalism [25]. The activation of the tip cell phenotype in endothelial cells is the result of their response to the environment through internal gene regulatory processes. This complex process is implemented in the model through an agent-based component. The phase-field model tracks the position of capillaries and the model parameters used (proliferation rate, cell velocity and diffusion constants) can be directly related to quantities measured experimentally (See Text S1).

In the model we introduce an angiogenic factor that represents the balance between pro and anti angiogenic signaling proteins [26] and that is produced by the tissue cells in hypoxia. We assume that these cells release angiogenic factor until oxygen is properly delivered to them. We further assume that new vessels follow the gradient of angiogenic factor and that their tip cells move with a velocity proportional to such gradient. When mentioned below, we consider in the model the dynamics of angiogenic factors which diffusion constants can be modified by events such as proteolysis. The capillary network morphology is analyzed for different levels of MMPs produced by the tissue cells, responsible for the control of the diffusion and availability of angiogenic factor.

We analyze the capillary network morphology for different values of the stalk endothelial cells proliferation rate and of the velocity of the tip endothelial cells in the tissue.

### Quick Guide to Equations and Assumptions

#### Equation 1 – Angiogenic factors

We describe the dynamics of an effective factor 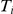 that represents the balance between pro and anti-angiogenic factors. The index indicates different angiogenic factors with different diffusive behaviors in the tissue, related to the presence of different VEGF isoforms. The angiogenic factor diffuses randomly from the hypoxic tumor area where it is produced. Schematically,
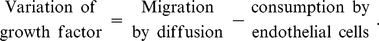
Mathematically, the equation for the time evolution of the concentration of 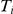, is given by
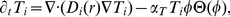
(1)where 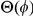 is the Heaviside function (the Heaviside function takes the value 1 when its argument is positive and zero otherwise), and 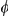 is the order parameter described below. We assume that the diffusion constant 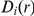 will primarily depend on the type of angiogenic factors considered: either diffusible isoforms for which 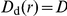 is constant, or heparin-binding isoforms for which 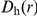 depends on position (see Text S1).

#### Equation 2 – Stalk cells

Proliferative and non-activated cells are described by an order parameter 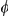 which is equal to 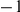 outside the capillary and 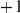 inside it. Values of 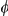 larger than one correspond to areas of high proliferation of endothelial cells which will lead to the widening of the capillary. The position of the capillary wall is given at the level set 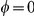. We assume that the dynamics of stalk endothelial cells is the result of the balance between the cell proliferation term, regulated by the concentration of angiogenic factor, and the term describing the dynamics of the vessel. We also assume that a well defined boundary exists between the vessel wall and the tissue. Thus,
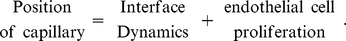
In the phase-field spirit, this can be mathematically written as
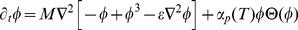
(2)where 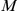 is the mobility coefficient for the endothelial cells and 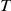 stands for the sum of the concentrations of all angiogenic isoforms considered. The proliferation rate 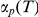 is given by 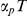 for 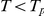, and is equal to its highest value 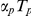 at concentrations of angiogenic factor higher than 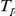. 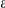 is the width of the capillary wall.

#### Equations 3 and 4 – Tip cells

The activated tip endothelial cell moves chemotactically with velocity **v** (proportional to the gradient of angiogenic factor, 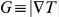) given by
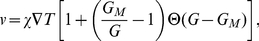
(3)where 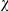 is the chemotactic response of the endothelial cells. The angiogenic factor at the tip cell is only consumed at its surface receptors, therefore we set 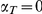 in equation (1) at all points inside the tip cell. The gradients entering equation (3) are measured at the center of the tip cell, and for gradients larger than 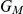 the tip cells attain their maximum velocity 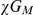. Note that while in equation (3) there is an abrupt functional transition at 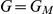, the model results do not depend crucially on the precise form of the function between square brackets in (3) (see Text S1).

In order for the endothelial tip cell activation to occur, points with large values for both 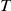 (the sum of the concentrations of all the isoforms of angiogenic factors) and 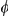 are necessary. In order for the activated cell to move, is also required a minimum value of the gradient of 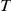, 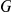. We assume that if these conditions are met at the center of the endothelial cell, its acquires the tip cell phenotype, with the caveat that cell-cell contact dependent mechanisms (as reported for the Notch pathway [27], [28]) prevent the activation of two neighboring cells. Inspired in this mechanism, only points for which there is a minimum distance of 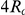 to the centers of all already existing tip cells can become centers of activated tip cells, where 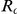 is the radius of an endothelial cell. We call 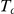 the minimum value of 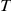 for tip cell activation, and 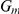 the minimum 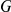 for the tip cell to move chemotactically [29] (in Text S1 we discuss in detail how these parameters are chosen in the simulation). As in the biological system, when the chemotactic signal is small, the endothelial cell returns to the stalk cell state, i.e. when 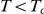 or 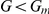.

To merge tip cell and capillary dynamics, we relate the value of the order parameter inside the tip cell 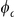 with the proliferation rate 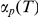 and the chemotactic response of the cells 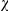 through:
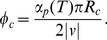
(4)This expression is obtained by calculating the excess cell density by proliferation occurring as the tip cell moves chemotactically in the tissue. The material produced in the region of the activated cell per unit time is 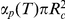, and the area per unit time that the cell sweeps is 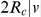. 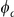 is obtained as the ratio of the material produced and the area swept. In our model, if the value of 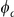 is smaller than 1, the capillary width becomes thinner than 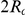 unless proliferating cells fill it in. On the other hand, if 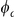 is larger than 1, the resulting capillary width becomes thicker than 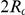. Hence, the value of 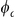 does not describe the density of cells at the tip cell, a meaningless concept *per se*, but the density of stalk cells on the vicinity of the tip cell.

## Results and Discussion

**Figure 1:**
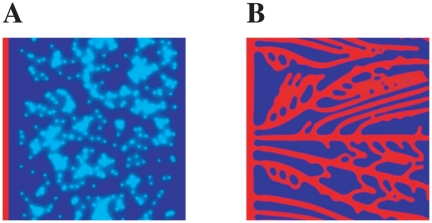
Growth of capillary network. Starting configuration presented in A. On the left side the initial vessel is represented in red. The concentration of the angiogenic factor produced by the hypoxic cells is plotted in light blue, and their location is denoted by the points where this concentration is higher. In B is the capillary network obtained for this distribution of hypoxic cells for maximum tip cell velocity and proliferation rate of 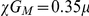
m/min and 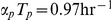 respectively.

Starting from a single vessel (at the left side in Figure 1A in red) close to a group of hypoxic cells (bright regions in Figure 1A) producing diffusible isoforms of angiogenic factor, a capillary network emerges. As tip cells are activated, they migrate towards the gradient of angiogenic factor (see Video S1). In Figure 1B we observe a hierarchical branched structure where small capillaries sprout from large ones. The overall alignment of the structure is directed towards the sources of angiogenic factor. There are several observed events of anastomosis which occur when two or more branches grow simultaneously in the direction of the same cell in hypoxia. We verified that the resulting capillary network is characterized by similar values of branch density and capillary diameter for different distributions of angiogenic factor sources (see Text S1).

### Chemotaxis vs Proliferation

We study the competing effects of tip cell velocity and the stalk cells' proliferation rate that determine the final morphology of the capillary network. For each case we measure the number of branches per mm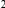 (a single branch comprises the capillary between two consecutive branching points) and the average vessel diameter.

**Figure 2:**
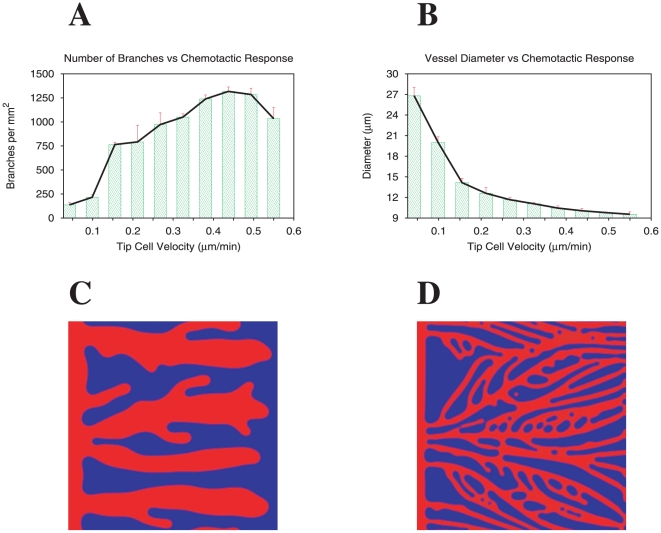
Capillary network and the tip cell's velocity. Branch density (A) and vessel diameter (B) as function of tip cell maximum velocity. Increasing the tip cell velocity leads to thinner vessels and a higher occurrence of branching points. Capillary network morphologies obtained for low (C) and high (D) tip cell velocities (respectively 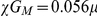
m/min and 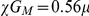
m/min). Proliferation kept constant at 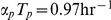.

We first vary the maximum tip cell velocity in the tissue and observe dramatic changes in the vascular pattern (by varying the parameter 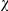 in the model). We verify that an increase of tip cell velocity leads to a higher branch density and to lower vessel diameter (Figures 2A and 2B). In Figures 2C and 2D we present two typical examples of the patterns observed at low and high tip cell velocities. For a low maximum velocity vessels are thick and little ramified (Figure 2C). Since the tip cell moves very slowly, there is time for stalk cell proliferation to occur and also for the stalk cells to consume angiogenic factor, keeping its concentration smaller than the concentration required for branching. The opposite occurs at high values of the tip velocity (Figure 2D). In this case the tip cells move rapidly, and the concentration of angiogenic factor at the stalk cells is high enough for ramification to exist. The vessels are thin and the structure very ramified.

**Figure 3:**
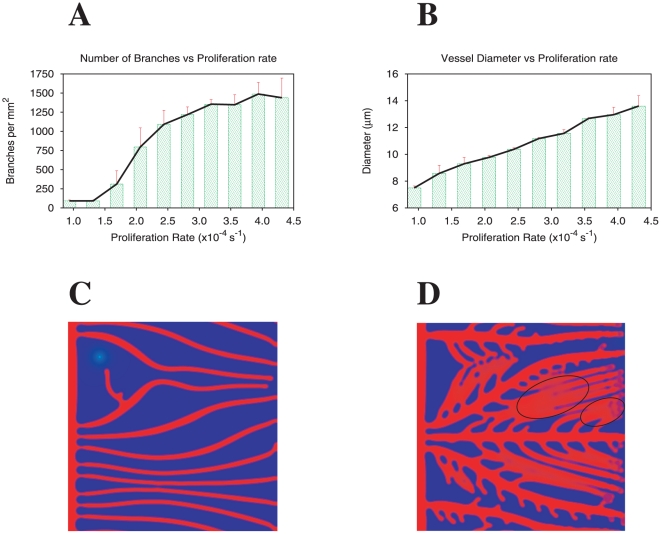
Capillary network and the stalk cell's proliferation rate. Branch density (A) and vessel diameter (B) as function of the stalk cell's maximum proliferation rate. Increasing the proliferation rate gives thicker vessels and a higher occurrence of branching points. The final capillary morphology can be controlled by careful tuning of the stalk cells proliferation rate and the tip cells velocity, which are functions of the chemical and physical environment of the tissue. Capillary network morphologies obtained for low (C) and high (D) proliferation rates (respectively 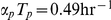, 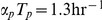). Ellipses mark merging events. Tip cell velocity kept constant at 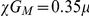
m/min.

We then vary the maximum proliferation rate of the stalk cells (by varying 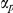 in the model) and observe that the increase of endothelial cell proliferation leads to an increase of the branch density and vessel diameter (Figures 3A and 3B). In Figures 3C and 3D we present two typical examples of the patterns observed at low and high proliferation rates. For low proliferation rates vessels are very thin and rarely thick enough for a tip cell to emerge, hence we observe very little ramification (Figure 3C). On the other hand, at high proliferation rates, vessels are thick and have a larger number of ramifications, and may merge laterally to form even thicker vessels (Figure 3D, ellipses mark merging events).

The values obtained in the model for the chemotactic velocity (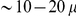
m/hr) agree with the ones observed experimentally (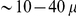
m/hr for cells in vitro [30]). The normalized endothelial cell proliferation coefficient (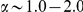) is moderately higher than the corresponding coefficient observed in vitro (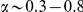 [31]). Notice however that the proliferation term in the model corresponds to the increase of vessel area per unit time. This description is ideal to be directly compared to observations of the network through diverse imaging techniques. Since the vessels are hollow, we expect that measurements of cell proliferation in vivo of the same order of magnitude but systematically below the cell proliferation term in the model. In order to compare directly the proliferation in the model to endothelial cell proliferation measured in vivo and in vitro, would be interesting to model the growth of hollow vessels within the framework presented in this work.

In summary, tip cell velocity and proliferation play important and complementary roles in the patterning of the vascular network. Both control the thickness and the ramification degree of vessels, but while a large tip cell velocity leads to thinner and more ramified vessels, a large proliferation rate leads to thicker and more ramified vessels.

It is remarkable that relatively small variations in magnitude of the parameters that regulate the tip cell velocity and proliferation in our model, lead to dramatic differences in the morphology of the vascular network. For example a factor of two in the proliferation rate leads to network morphologies as different as the ones shown in Figures 1B and 3C. This suggests that angiogenesis is the result of a delicate balance between chemotaxis and proliferation (as suggested in the literature [32], [33]), and that variations in the parameters that determine these two effects could be responsible for the differences observed in the vascular patterns of different tissues and pathological conditions.

These results provide a novel perspective concerning the causes that determine the various vascular patterns, which have been normally considered to be determined only by soluble and matrix-bound growth factors (such as VEGF) [23]. We verify that the constitution of the ECM has a direct implication on the ability of the endothelial cells to move through it, thus regulating their velocity. Also a different composition of the angiogenic chemical cocktail may lead to different proliferation rates of the endothelial cells. We conclude that the resulting capillary network morphology is not only a result of the levels and distribution of angiogenic factor, but also of the extracellular matrix properties and the exact composition of the protein cocktail.

### Comparison with experimental results

We test our mathematical model against two experimental situations.

#### Morphology dependence on the amount of angiogenic factor

First, we compare with results obtained by Haigh et al [33], that report differences in vascular patterning due to an angiogenic factor dose-dependent effect. This manuscript describes several in vivo mouse models in which the expression of VEGF-A is selectively regulated by the use of tissue-specific promoter-driven transgene technology. These authors approached the visualization of the vascular network in the developing cortex and retina of embryos and newborn mice. The reported results on the effects of intermediate and severe reductions of VEGF-A levels in the vasculature represent a valid tool to test our theoretical modeling.

Although the final goal of these authors was to determine the role of VEGF-A during the development of the nervous system, their neat approach allowed the visualization of the vascular network in the developing cortex and retina of embryos and newborn mice. The authors reported that the decrease of VEGF-A is accompanied by a sparser vessel network when compared to the wild type used as a control condition (see for example 3D reconstructions of the capillary vasculature of the retina of 21 days old mice in figures 2G and 2H from [33]). Surprisingly, the thickness of the vessels does not vary.

**Figure 4:**
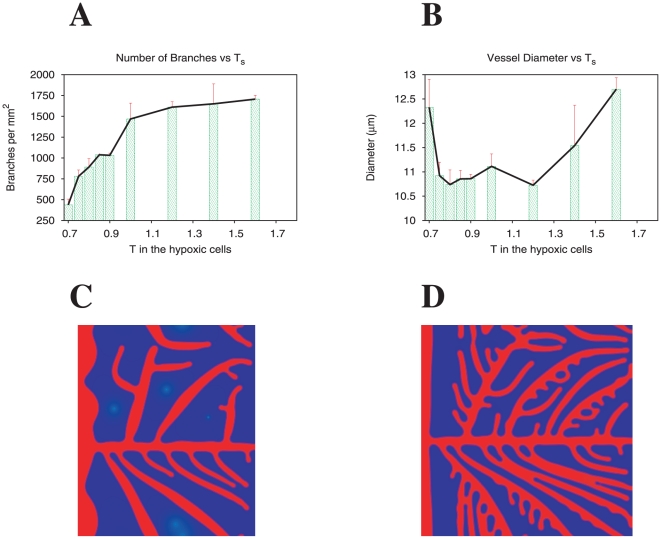
Morphology dependence on the amount of angiogenic factor (comparison with experimental results). Quantification of branch density (A) and vessel diameter (B) as function of the angiogenic factor production levels of the hypoxic cells. Capillary network morphologies modeled for lower (C) and higher (D) levels of angiogenic factor (respectively 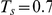 and 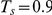). For a relatively wide range of angiogenic factor production levels (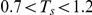, an increase of these levels lead to an increase in vessel density but not to an alteration of vessel diameter.

We approached this condition by simulating the capillary vessel network formation in the presence of different levels of angiogenic factor produced by the tissue cells. In Figures 4C and 4D we present two typical examples of the patterns observed at low and intermediate levels of factor production. Qualitatively comparing these experimental and theoretical images we observe that the lower the angiogenic factor concentration, the sparser the vessel network that is obtained. In addition we do not observe appreciable changes in the thickness of the vessel structures in both experimental and theoretical situations.

In order to obtain more quantitative predictions of the model, we study the variation of branch density and vessel diameter for different amounts of angiogenic factor present (Figures 4A and 4B). We verify that there is a relatively large region of angiogenic factor concentration in which an increase of the angiogenic factor leads to higher branch density but not to an appreciable variation of the vessel diameter. In fact, even at larger amounts of angiogenic factor, where vessel diameter increases with respect to the values at the plateau, the observed diameters are between 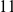 and 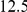 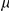
m thus presenting a rather small variation of vessel diameter.

Our model also predicts that at very low values of angiogenic factor, the vessel network cannot be formed (for 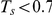 in this case). This agrees with the experimental observation that a severe reduction of VEGF-A production levels leads to the lack of a functional network that causes early embryonic lethality, exemplified by the lack of a functional network [33]–[35].

On the other hand, VEGF over-expression has also been addressed experimentally in different manners [36]–[38] leading invariably to the presence of thicker vessels. This fact is also observed in the model at high levels of angiogenic factor we observe lateral merging of thin vessels leading to thicker capillaries (see Figure S1).

The predicted values of our model for the vessel density, agree well with the ones measured by Hellström and colleagues [28] (where the authors vary the activity of the Notch pathway during the development of mice retina). They observe vessel diameters between of 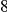 to 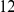 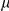
m and around 600 to 1200 branching points per mm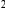 (see for example Figure 2 of [28] and its Supporting Information), which agrees well with the diameters and branch densities observed in this simulation of mouse retina vasculature.

#### Morphology dependence on alteration of the balance diffusible/bound angiogenic factor

In our attempt to reproduce further complex situations, we address the results by Rodr guez-Manzaneque *el al.* [39], whose mouse models involve the progression of mammary gland tumors under the influence of altered activity of MMPs.

MMPs have capacity to mobilize heparin-binding VEGF isoforms from their reservoirs in the ECM, affecting their diffusion constant which will be a function of the distance to the MMPs producing cells [23]. The study presented in [39] reports the generation of distinct vascular patterns as a result of changes in the bio-availability of VEGF, dependent of MMPs activity (see also [23]). In particular were observed mammary tumor-prone mice that either lack, or specifically over-express Thrombospondin-1 (TSP1) in the mammary gland. TSP1 is an inhibitor of MMPs activation, and so, the under-expression of TSP1 leads to an increase in the MMPs activity and a larger bio-availability of VEGF. Hence TSP1 is a anti-angiogenic factor [40]. Conversely, it was observed that over-expressors of TSP1 (low MMPs activity) showed less frequent tumor capillaries with reduced diameter, and a sinuous phenotype, while vasculature significantly increases in TSP1-deficient (high MMPs activity) animals with thicker, frequent and straighter vessels (see for example figures 3F and 3I from [39]).

We introduce two distinct forms of angiogenic factors in the model. Although the identity of MMPs-producing cellular entities includes most of the tumor-associated cells, we consider the hypoxic cells in the tumor microenvironment as those able to produce MMPs, affecting the diffusion of the heparin-binding angiogenic factor isoforms in their surroundings (we present in Text S1 the derivation of the dependence on the position of the heparin-binding isoform's diffusion constant). The concentration of the heparin-binding isoforms at the tumor cells, is set equal to the concentration of the diffusible isoforms, 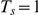.

In the following simulations we investigate the final capillary network morphology for different values of MMPs produced by the tissue cells. In our model we vary the radius of the region around each hypoxic cell where MMPs activity is high, with the consequence of releasing from the ECM the heparin-binding isoforms of angiogenic factor. In what follows the chosen values for maximum proliferation rate and maximum tip cell velocity are 0.69 hr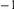 and 0.35 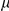
m/min respectively.

**Figure 5:**
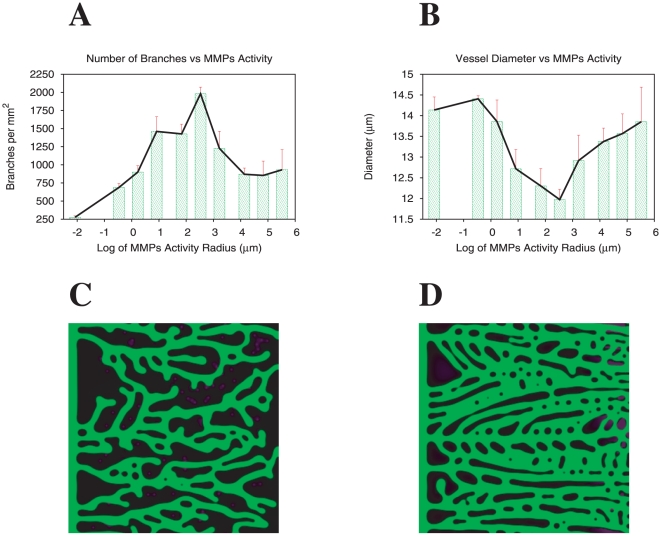
Morphology dependence on alteration of the balance diffusible/bound angiogenic factor (comparison with experimental results). Quantifications of branch density (A) and vessel diameter (B) as function of the MMPs activity radius. At low activity of MMPs is observed the localization the heparin-binding angiogenic factor isoforms close to the hypoxic cells, leading to a network characterized by thin and sinuous vessels. On the other hand, a release of the ECM bound angiogenic factor by MMPs leads to lateral merging of vessels generating thicker and straighter vessels. Capillary network morphologies modeled for low (C) and high (D) levels of MMP activity (MMP activity radius respectively 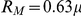
m and 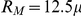
m; simulation figures in green and black for better comparison with experimental results).

We verify that at low values of MMPs activity, vessels are few and sinuous in clear agreement with experimental results. This is clearly visualized in Figure 5C. The localized presence of high concentrations of heparin-binding angiogenic factor orients tip cells in the direction of neighboring cells in hypoxia. The directing gradient reflects the local anisotropies of the tissue, and the capillary network is a result of these local gradients.

At high values of MMPs activity, the number of branches is smaller and the branch width is larger. Many thin vessels merge (Figure 5D), leading to the experimentally observed thick vessel phenotype [39]. Note that vessel merging is also observed in other in vivo models with large bio-availability of VEGF [37].

In Figures 5A and 5B we report systematically the observed branch density and capillary diameter as a function of the logarithm of the MMPs activity radius. As the MMPs activity varies we observe a non-monotonic behavior for both, the number of branches and the vessel diameter. In an intermediate regime of MMPs activity values, the release of the heparin-binding angiogenic factor isoforms from the neighborhood of the hypoxic cells, leads to a strong gradient of angiogenic factor at the tip cells, which directs them towards the hypoxic region, but not to any cell in particular. The result is a straighter vessel phenotype. Since the vessels arrive to their destinations faster, (as they are directed by a well defined gradient) there is less time for proliferation to occur and vessels become thinner. At higher values of MMPs activity the thin vessels merge leading to an increase of average vessel diameter in the system, and decrease of vessel density (Figures 5A and 5B).

We verify that by varying the amount of MMPs the tissue may have a very precise control over the type of capillary network formed.

### Conclusion

In summary, the model presented reproduces in vivo patterns of newly formed vascular networks. This approach has the benefits of a low-number of parameters formulation and the capability of producing in silico vascular networks able to be inspected from a morphological point of view.

The model simulates the main mechanisms responsible for vascular patterning. The resulting network is solely a function of the diffusion properties and levels of the angiogenic factor, and of the migration velocity and proliferation rate of the endothelial cells. The other model few parameters are fixed after the endothelial cell size and oxygen diffusion length in the tissue are identified (see Text S1).

For each of the analyzed cases, we study how the resulting vasculature is quantitatively altered when the respective parameter is varied. We verified that an increase in proliferation leads to a more ramified network constituted by thicker vessels, and a higher migration velocity leads to a more branched network with thinner vessels. We also observed that these alterations take place in a relatively small range of parameters, indicating that a precise regulation of these characteristics is essential to define the final vascular pattern.

To verify experimentally this is the case, we suggest that specific molecules with very well defined role in either the pathway regulating chemotaxis or the pathway regulating proliferation in endothelial cells, could be used to probe the influence of each one of these factors independently. Other possible experiment would be to use a Matrigel plug assay to characterize the vasculature for different levels of added collagen, which would alter the ability of endothelial cells to move in the matrix but not their proliferation rate. We suggest that the simulation of growth of hollow tubes within the phase-field framework would be of interest to improve the correlation between endothelial proliferation and capillary volume growth.

With respect to the levels of angiogenic factor in the tissue, we showed that a higher production increases the branch density sharply, and finally vessels start merging, leading to a higher vessel diameter. An increase in the bioavailability of angiogenic factor, on the other hand, leads first to a larger number of thinner vessels that, at even higher levels of MMPs, merge forming thicker vessels. These are non trivial dependencies on the parameters that are verified experimentally and captured by the model.

The model presented in this paper can also be used in many more experimental setups to validate and suggest different biological hypothesis. This sort of systematic theory/experimental comparison requires an exhaustive work coming from both sides and will be the aim of a future work. As an example of possible different situations, in Figures S2, S3 and S4 we plot capillary networks for different hypoxic cell densities and distributions, as well as for the case where Notch signaling is defective. In particular in this last point we obtain a qualitative agreement with experimental verification [28], though the model could be improved by including a more complete description of regulation of tip cell activation mediated by Dll4-Notch signalling.

During the last decades, the understanding of cancer biology in general, and of tumor angiogenesis in particular, has been favored by the convergence of various disciplines. While the use of genomic and proteomic platforms has been consolidated, and in fact they form part of an extensive number of translational units and oncological research departments, the field of in vivo imaging is an emerging challenge where more efforts need to be directed. Recent progresses on high resolution microscopy [10] have given the hope that cancer progression can be monitored, at least regarding the characteristics of its associated vasculature. The concurrence of sophisticated image-tracking systems and advanced mathematical models, like the one presented here, will provide a relevant tool to perform early prediction and diagnosis, and can help to define the proper therapies to be followed.

## Supporting Information

Figure S1
**Capillary network morphology obtained for very high production levels of angiogenic factor by the tissue cells.** The observed vessels are straight and thick due to high endothelial cell proliferation and lateral vessel fusion (also verified experimentally [36]). The value for 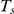 used in this simulation is 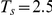.(EPS)

Figure S2
**Capillary network morphology obtained for different number of angiogenic factor sources.** Figures A, B, C, and D have respectively 120. 240, 480 and 960 initial hypoxic cells approximately. At low density of sources (Figures A and B) the network forms a tree-like structure, with a low number of branches, similar to the network at low values of 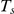 (see Figure 4C). As the density of hypoxic cells increase (Figures C and D) the gradient of 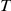 becomes perpendicular to the main capillary and many sprouting events occur, leading progressively to a straighter vessel phenotype. Notice that this case is different from a high 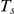 situation. While the two scenarios have high 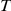 levels, in the case the number of hypoxic cells is large, the variations of the 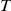 gradient in space are much lower.(EPS)

Figure S3 **Capillary network morphology obtained for a large initial circular source of angiogenic factor.** Figures A, B, C, and D are snapshots of the vasculature growth. The resulting network is tree-like and very dense when reaches the source. This is a brush-like network as observed in various solid tumor situations (both in silico and in vivo) [14], [41].
(EPS)

Figure S4
**Capillary network for deficient Notch signaling (considering length for tip cell activation 2** 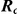 **instead of** 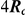 **, measured between cell centers, hence allowing for two adjacent cells to acquire both the tip cell phenotype).** The figures represent the observed capillary network morphology for low and high proliferation rates; respectively 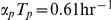 (A and C) and 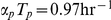 (B and D). In figures A and B there is deficient Notch signaling while C and D present the corresponding vascular patterns for functioning Notch signaling. A deficient communication between neighboring cells leads to a larger number of ramifications which are able to merge laterally, giving a higher average vessel diameter. This is in qualitative agreement with experimental verification by Hellström et al (see figure 1 of Supporting Information in [28]).(EPS)

Text S1 **Supporting information with details on the model equations.**
(PDF)

Video S1
**Formation of the capillary network shown in** **Figure 1B** **.** The tip cell velocity and stalk cells proliferation rate in this simulation are 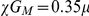/min and 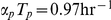 respectively.(MOV)
