# Correction: Tumor Angiogenesis and Vascular Patterning: A Mathematical Model

**DOI:** 10.1371/annotation/53aa27c5-0c32-4904-9278-4a68c39963d2

**Published:** 2011-06-06

**Authors:** Rui D. M. Travasso, Eugenia Corvera Poiré, Mario Castro, Juan Carlos Rodrguez-Manzaneque, A. Hernández-Machado

The 4th author's name is misspelled. The correct spelling is: Juan Carlos Rodríguez-Manzaneque

The correct citation is: Travasso RDM, Corvera Poiré E, Castro M, Rodríguez-Manzaneque JC, Hernández-Machado A (2011) Tumor Angiogenesis and Vascular Patterning: A Mathematical Model. PLoS ONE 6(5): e19989. doi:10.1371/journal.pone.0019989

