# Correction: Tumor Angiogenesis and Vascular Patterning: A Mathematical Model

**DOI:** 10.1371/journal.pone.0146650

**Published:** 2016-01-05

**Authors:** Rui D. M. Travasso, Eugenia Corvera Poiré, Mario Castro, Juan Carlos Rodrguez-Manzaneque, A. Hernández-Machado

The following information is missing from the Funding section: This work was also supported by Fundos FEDER through Programa Operacional Factores de Competitividade COMPETE through the project with reference number FCOMP-01-0124-FEDER-015708.
