# Supplementary material for: Tumor Angiogenesis and Vascular Patterning: A Mathematical Model
Source: PLoS One. 2011 May 27;6(5):e19989. doi: 10.1371/journal.pone.0019989 (PMC3103509; doi:10.1371/journal.pone.0019989)
Supplement: Text S1 — Supporting information with details on the model equations. (PDF) [file pone.0019989.s005.pdf]

Text S1

The equations and the phase-field model

The model introduces a hypoxic cell produced angiogenic factor. This factor diffuses in the tissue and is consumed by the capillary endothelial cells. These proliferate in the presence of the angiogenic factor and may acquire the tip cell phenotype. Tip cells move in the direction of the gradient of angiogenic factor. These processes are described by the following equations:

$$\partial_t T_i = \nabla \cdot (D_i(\mathbf{r}) \nabla T_i) - \alpha_T T_i \phi \Theta(\phi) \ , \tag{1}$$

$$\partial_t \phi = M \nabla^2 \left[-\phi + \phi^3 - \epsilon \nabla^2 \phi \right] + \alpha_p(T) \phi \Theta(\phi) \ , \tag{2}$$

$$\mathbf{v} = \chi \nabla T \left[1 + \left(\frac{G_M}{G} - 1 \right) \Theta(G - G_M) \right] \ , \tag{3}$$

$$\phi_c = \frac{\alpha_p(T) \pi R_c}{2|\mathbf{v}|} \ . \tag{4}$$

Equation (1) describes the diffusion of T_i in the tissue and its consumption by the endothelial cells. Equation (2) describes the capillary-tissue interface dynamics and the stalk cell proliferation. Equation (3) gives the tip cell velocity and equation (4) gives the value of the order parameter inside the tip cell. A new tip cell is activated where $\phi > 0.9$, $T > T_c$ and $|\nabla T| > G_m$ if two distance requirements are satisfied. Firstly, the center of the new cell must be located further than a cell radius length from the surface of the capillary, and secondly it must also be located at least two diameters away from any other tip cell center (due to the Notch signaling). At most one cell is activated per integration time step. Biologically, the mechanism for Notch signaling in tip cell activation is only relevant for cells belonging to the same branch. In the systems analyzed, we find that the positions where tip cell sprouting may occur are located where the concentration of angiogenic factor is higher, i.e. just behind already existing tip cells. Consequently, and in spite of not imposing it explicitly in the code, we verify that in each capillary the inhibition of sprouting through tip cell proximity (akin to the function reported for the Notch signaling pathway) is always regulated by the capillary's own tip cell. In fact, because of the short distance of inhibition, $4R_c$, and because the points able to branch are few, a tip cell of another capillary is not able to be closer to the sprouting points of a particular branch than the branch's own tip cell.

The sources of T_i are distributed in the tissue randomly. The concentration of angiogenic

factor at each source is set to the constant value $T_i = T_s$ until a capillary exists at a distance closer than d, the typical diffusion distance of oxygen in the tissue [1] (oxygen diffusion is considered instantaneous in the time scale of capillary development). Were also simulated the development of capillary networks for regular distributions of sources of angiogenic factor sources representing the regular arrangement of cells in the tissue. We verified that the resulting capillary network is characterized by similar values of branch density and capillary diameter as in the random case. Nevertheless we verified a more regular distribution of major vessels (see Figure 1). In the case of a square lattice of T sources the first sprouting vessels are parallel to each other, while in a triangular lattice of T sources we observe some such vessels directed along angles close to 30 and 60 degrees, hence following the trend of the underlining lattice.

In equation (1) the T_i consumption factor α_T is given as $\alpha_T = D/R_c^2$, where R_c is the radius of the endothelial cells. The reason for this dependence is that the capillary wall acts as a barrier for the angiogenic factor, and therefore its concentration decays within the length scale on the order of the cell size. $\alpha_T = D/R_c^2$ is obtained from the stationary solution of equation (1) close to the capillary boundary.

Equation (2) describes the evolution of the field ϕ which characterizes the phase of the system. The second term of this equation represents the proliferation rate. The first term is on the form of a conservation equation:

$$\partial_t \phi = -\nabla \cdot \mathbf{j} \ , \tag{5}$$

with

$$\mathbf{j} = -\nabla \mu \ , \quad \mu = \frac{\delta F[\phi]}{\delta \phi} \tag{6}$$

where μ is the chemical potential related to $F[\phi]$, a free energy functional of the order parameter ϕ. In this case we take the Ginzburg-Landau free energy functional

$$F[\phi] = \int \left(-\frac{\phi^2}{2} + \frac{\phi^4}{4} - \frac{\epsilon}{2} |\nabla \phi|^2 \right) \mathrm{d}\mathbf{r} \ . \tag{7}$$

The Ginzburg-Landau free energy (7) implements an energy cost proportional to ϵ per unit length of interface. Therefore the dynamics is driven by the minimization of the interfacial length. At the same time ϵ represents the interface width of the capillaries.

Obtaining quantitative results

Equations (1)-(4) are written with dimensionless quantities. In order to obtain quantitative information from the simulation, we have to relate the magnitude of the different constants with observed values *in vivo*.

Endothelial cells are a very diverse population [2] taking distinct functions and forms in different circumstances. Here we fix the lattice unit size in the simulation to be equal to 1.25μm and the value for R_c as $R_c = 5\mu$m = 4 lattice units [3]. The diffusion constant of the chemo-attractants is typically two to three orders of magnitude higher than the diffusion constant associated with the endothelial cell movements [4]. In the model the ratio between these two diffusion constants is equal to $D = 100$. The mobility of the endothelial cells, set as $M = 1$ in equation (2), may vary considerably with the type of tissue. Considering it to be on the order of 10^{-11} cm^2/s [4] fixes the time unit of the simulation in 26 min.

The typical oxygen diffusion size used in the simulation is 2.5 cell diameters ($d = 25\mu$m) in agreement with the literature [1]. The simulation box used is 375×375 μm. The time taken for each network to be formed in this simulation box varies between 50 and 500 time units, depending on the value of χ, which we vary within an order of magnitude. Therefore, in these situations the new vasculature takes 22 hours to 9 days to cover the square of 0.14 mm^2, depending on the type of network formed and starting from a single capillary.

When varying the chemotactic response χ and endothelial cell proliferation α_p, we are observing the formed network for different values of the maximum tip cell velocity χG_M and maximum proliferation rate $\alpha_p T_p$ respectively. Notice that all parameters in the simulation can be directly matched to experimental values except for the ones related with the magnitude of the angiogenic factor (as χ or α_p), since T_i represents an average effect of the different factors in the system. Nevertheless the products χG_M and $\alpha_p T_p$ do not depend on the T_i units and can be easily quantified. Inserting the values of G_M and T_p used in the simulation, the maximum tip cell velocity is equal to 1.4χ nm/min and the maximum proliferation rate is equal to 0.69α_p hr^{-1}. We observe in the simulations presented that a functional network is formed for $\chi = 250$, corresponding to a maximum tip cell velocity of 0.35 μ/min. The effective diffusion constant of tip cells, with radius 5 μm, comes on the order of 3×10^{-10} cm^2/s, close to the value used in [4].

For more details on the parameters used, please refer to the next section and to Table 1.

The equations are integrated using a finite differences integration scheme. The program execu-

tion follows the flowchart in Figure 2.

Tip cell parameterization

For tip cell activation to occur, the value of T_c has an upper limit. In the model angiogenic factor sources (hypoxic cells) are at at a distance larger than d from a capillary. To estimate the upper limit for T_c we consider a source at $x = d$ (where $T = T_s$), the capillary wall at $x = 0$, and solve the following equations in one dimension:

$$\partial_t T = D\partial_x^2 T \ , \qquad x \geq 0 \ ,$$
$$\partial_t T = D\partial_x^2 T - \alpha_T T \ , \quad x < 0 \ .$$

These equations are the simplification of (1) for a very sharp interface. Finding the steady state solution for T (continuous at $x = 0$) we obtain

$$T(x) = \begin{cases} \frac{T_s}{1+d/R_c} + \frac{Ts}{R_c+d}x \ , & x \geq 0 \\ \frac{T_s}{1+d/R_c}e^{x/R_c} \ , & x < 0 \end{cases} \ , \tag{8}$$

where $R_c = \sqrt{D/\alpha_T}$ is the cell size. For tip cell activation, the value of T at its center must be larger than T_c, so

$$T_c \lesssim \frac{T_s e^{-1}}{1 + d/R_c} \ . \tag{9}$$

Since $d = 25$ μm and $R_c = 5$ μm, for $T_s = 1$ we estimate that in the stationary case, the value of T at the center of the cells is $T \approx 0.061$. The value chosen for tip cell activation in the code, $T_c = 0.055$, is close to this estimated value. If T_c were much below this value it would lead to a very large number of activated cells, while a value of T_c much larger would result in no tip cell activation. Also, in the same way as in nature, a very low production level of angiogenic factor (low T_s) does not promote tip cell activation [5, 6], since inequality (9) stops being valid.

At large concentrations of VEGF, however, tip cell migration may be impaired when the gradient of VEGF is very shallow [7]: the capillary endothelial cells present filopodia but the gradient is not strong enough to direct their migration. Since in this case, the tip cell does not perform its role as an agent leading capillary growth, the model will consider there was no tip cell activation, and capillaries become thicker by proliferation only [7]. The minimum gradient of T for tip cell activation used in the model is the gradient at which $\phi_c \approx 1$, using equation (4) in a well formed network

(for example, with the parameters considered in Figure 1B of the main text). Approximating T by the expression (8) obtained for the steady state, ϕ_c is given by

$$\phi_c = \frac{\alpha_p \pi R_c}{\chi G_m} \frac{T_s}{1 + d/R_c} \approx \frac{0.015}{G_m} \ ,$$

where in the last step α_c, χ, R_c, d and T_s were replaced by the values used in Figure 1B of the main text. In the model we choose $G_m = 0.0125$ a.u./μm, (giving $G_m = 0.01$ in simulation units; notice that the angiogenic factor concentration is measured with respect to the production levels at the hypoxic cells), so that $\phi_c \approx 1$.

At large angiogenic factor gradients the velocity of the tip cell is limited by the tissue properties and the saturation of active angiogenic factor receptors at the front of the cell [8] (where the concentration of angiogenic factor is higher). We consider this fact in the model and take χG_M as the maximum tip cell velocity. In Figure 3 we present the observed network for different values of G_M (the other parameters are the same as in Figure 1B of the main text). We verify that the resulting vasculature characteristics (branch density and diameter) do not depend strongly on the value of G_M.

Extending the model to describe different angiogenic factor isoforms

The binding of VEGF to the ECM and its cleavage by MMPs has been studied carefully, leading to the prediction of different regimes for the concentration of heparin-binding and diffusible VEGF [9].

The angiogenic factor interaction with the ECM in included through a minimal model which captures the basic physics of the system. As T_i represents a balance between pro-angiogenic and anti-angiogenic factors, the difference in ECM binding affinity between the different factors, is represented by distinguishing two types of T_i, namely T_d and T_h, from diffusible and heparin-binding isoforms. T_d dynamics is identical to the one described by equation (1) but since T_h is able to bind to the ECM, the diffusion constant in (1) will have to be altered.

Consider the relation between the concentration of free (c_f) and bound (c_b) heparin-binding angiogenic factor isoforms to follow a Michaelis-Menten relationship [9]

$$c_b = \frac{C c_f}{K + c_f} \ .$$

Here K is the angiogenic factor dissociation constant [10] which is a function of the local concentration of MMPs [11], and C is the concentration of binding sites at the ECM. The diffusion equation for $T_{\rm h} = c_f + c_b$ is:

$$\partial_t T_{\rm h} = D \nabla^2 c_f \ .$$

Using the Michaelis-Menten relation we express c_f as a function of $T_{\rm h}$. For small gradients of $T_{\rm h}$ the previous expression can be written as

$$\partial_t T_{\rm h} = D_{\rm h} \nabla^2 T_{\rm h} \ ,$$

with

$$D_{\rm h} = \frac{D}{2} \left(1 + \frac{K - C + T_{\rm h}}{\sqrt{(K + 0.7 - T_{\rm h})^2 + 4 K T_{\rm h}}} \right) \ .$$

The parameters K and C depend on the characteristics of the ECM. Here we consider that the maximum concentration of bound angiogenic factor is $C = 0.7$ of the amount of factor produced by the tumor cells ($T_s = 1$). We also consider K to be directly proportional to the concentration of MMPs.

MMPs are produced by the tumor cells, diffuse in the ECM (diffusion constant D_M) and have a half-life γ_M. Therefore in the neighborhood of a tumor cell, the concentration of MMPs, c_m, is given by

$$c_m \propto e^{-r/R_M} \ ,$$

where r is the distance to the cell and $R_M = \sqrt{D_M/\gamma}$ is the diffusion radius of MMPs. The distance R_M measures the radius around each hypoxic cell for which the effect of MMPs on allowing the diffusion of the heparin-binding isoforms of the angiogenic factor is important. Hence R_M is the parameter regulating the activity of MMPs produced by the tumor cells.

In this model we use the dependence for K in R_M in a way to explore both the regime where the angiogenic factor is bound (with $D_{\rm a} \approx 0$) as well as when it is free (with $D_{\rm a} \approx D$). Implementing

$$K = 10 e^{-r/R_M} \ , \tag{10}$$

leads to finding $D_{\rm h}$ in the range from zero to $0.94D$.

Simulating directly the diffusion of MMPs and considering instead of (10), $K = 10 c_m$, yields

no appreciable change in network morphology (see Figures 4A and 4B). Also including in the model tip cell MMPs production and its role in freeing angiogenic factor from the matrix does not alter the resulting network characteristics (see Figures 4C and 4D). We conclude that the introduction of these two mechanisms in the simulation is not essential to obtain the correct vasculature parameters.

In the same way as for the diffusible isoforms, the concentration of the heparin-binding isoforms at the hypoxic cells is equal to $T_s = 1$ until their production is halted when a capillary is at a distance smaller than d from the cell.

References

[1] Grote J (1983) *Tissue Respiration*, in *Human Physiology*, eds Schmidt RF, Thews G (Springer-Verlag, Berlin) pp 508-520.

[2] Conway EM, Carmeliet P (2004) *The diversity of endothelial cells: a challenge for therapeutic angiogenesis.* Gen. Biol. 5:207.

[3] Gebb S, Stevens T (2004) *On lung endothelial cell heterogeneity* Microvascular Res. 68:1-12.

[4] Schugart RC, Friedman A, Zhao R, Sen CK (2008) Wound angiogenesis as a function of tissue oxygen tension: A mathematical model. *Proc. Natl. Am. Sci. USA* 105:2628-2633.

[5] K. Bentley, H. Gerhardt, P.A. Bates (2008) Agent-based simulation of Notch-mediated tip cell selection in angiogenic sprout initialisation. *J. Theo. Biol* 250:25-36.

[6] Haigh JJ, Morelli PI, Gerhardt H, Haigh K, Tsien J et al (2003) Cortical and retinal defects caused by dosage-dependent reductions in VEGF-A paracrine signaling. *Dev. Biol.* 262:225-241.

[7] Gerhardt H, Betsholtz C (2005) *How do endothelial cells orientate?*, in *Mechanisms of Angiogenesis*, eds Clauss M, Breier G. (Birkhauser, Basel), pp 3-16.

[8] Barkefors I, Le Jan S, Jakobsson L, Hejll E, Carlson G et al (2008) Endothelial cell migration in stable gradients of vascular endothelial growth factor A and fibroblast growth factor 2: Effects on chemotaxis and chemokinesis. *J. Cell. Biol.* 283:13905-13912.

[9] Small AR, Neagu A, Amyot F, Sackett D, Chernomordik V et al (2008) Spatial distribution of VEGF isoforms and chemotactic signals in the vicinity of a tumor. *J. Theo. Biol.* 252:593-607.

[10] Horn AS (1979) *Characteristics of dopamine uptake*, in *The Neurobiology of Dopamine* ed Horn AS, Korf J, Westerink BHC (Academic Press, London) pp 217-235.

[11] Lee S, Jilani SM, Nikolova GV, Carpizo D, Iruela-Arispe ML (2005) Processing of VEGF-A by matrix metalloproteinases regulates bioavailability and vascular patterning in tumors. *J. Cell Biol.* 169:681-691. tumor-induced angionenesis. *Annu. Rev. Biomed. Eng.* 8:233-257.

Figure Captions

Figure 1: Capillary networks formed in on regular arrays of hypoxic cells. Original distributions of hypoxic cells (A, squared lattice and C triangular lattice) and observed capillary networks for the same parameters as used in Figure 1B of the main text (B and D respectively) evidence similar branch density and vessel diameter are similar to the ones observed in Figure 1B of the main text.

Figure 2: Code's flowchart.

Figure 3: Capillary network morphology obtained for different values of G_M. For A, B, C and D the value for G_M is respectively $G_M = 0.025$, 0.0375, 0.0625 and 0.125 a.u./μm. The resulting network characteristics (branch density and diameter) do not depend strongly on this parameter.

Figure 4: Capillary network morphology obtained for different implementations of MMPs dynamics. In A we present the network obtained for $R_M = 0.5$ μm with the model used in the rest of the article. In B we show the network obtained in the same conditions but considering explicitly the diffusion of MMPs. The characteristics of the network are not altered. In Figures C and D we include tip cell production of MMPs. The concentration of MMPs at the tip is 0.2 and 20 times the concentration at the hypoxic cells for C and D respectively. This change in two orders of magnitude does not bring appreciable changes in the capillary network branch density and branch diameter. We conclude that the introduction of these two mechanisms is not essential to the final results.

Table 1

Parameter	Meaning	Value in Simulation	Value in vivo
	Lattice unit	1	1.25 μm
	Time unit	1	26 min
R_c	Cell radius	4	5 μm [3]
D	Ang. factor diffusion constant	100	10^{-13} m^2/s [4]
M	End. cell Mobility	1	10^{-15} m^2/s [4]
d	Oxygen diffusion length	20	25 μm [1]
$\alpha_T = D/R_c^2$	Ang. factor consumption rate	6.25	0.004 s^{-1}
ϵ	Interface width	1	1.25 μm
T_s	Ang. factor conc. at source	1	
T_p	Ang. factor conc. for highest proliferation	0.3	
T_c	Ang. factor conc. for branching	0.055	
G_M	Ang. factor grad. for highest velocity	0.03	0.0375 a.u./μm
G_m	Ang. factor grad. for branching	0.01	0.0125 a.u./μm

Table 1: Values of the parameters used in the simulations.

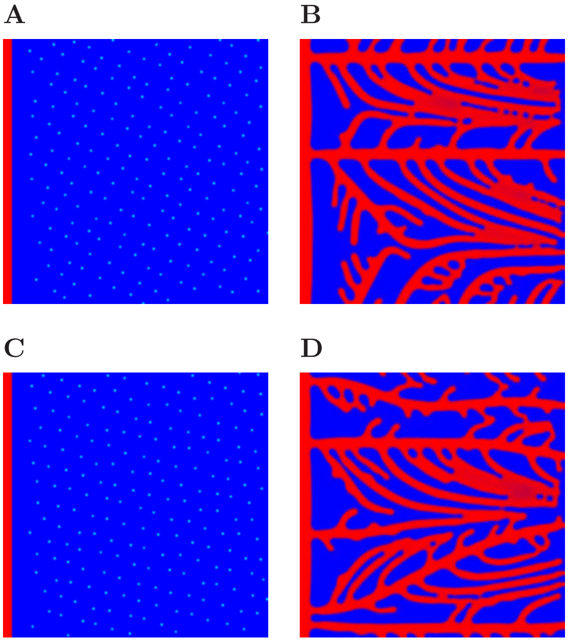

Figure 1

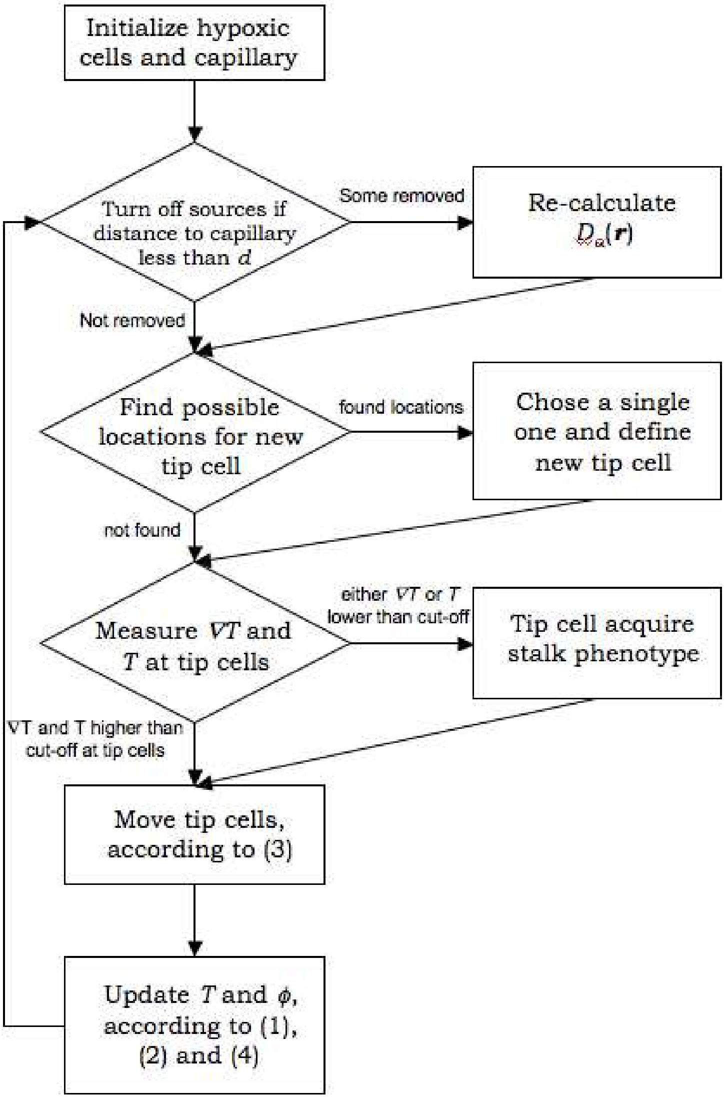

Initialize hypoxic cells and capillary
Turn off sources if distance to capillary less than d
Some removed
Re-calculate $D_\alpha(\mathbf{r})$
Not removed
Find possible locations for new tip cell
found locations
Chose a single one and define new tip cell
not found
Measure ∇T and T at tip cells
either ∇T or T lower than cut-off
Tip cell acquire stalk phenotype
∇T and T higher than cut-off at tip cells
Move tip cells, according to (3)
Update T and ϕ, according to (1), (2) and (4)

Figure 2

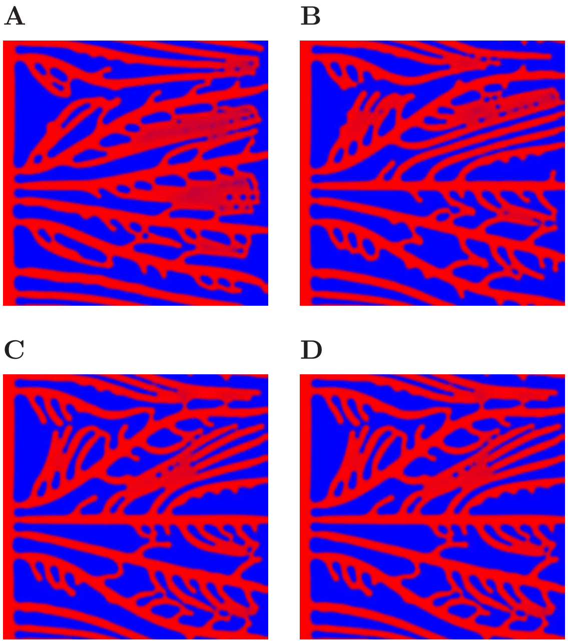

Figure 3

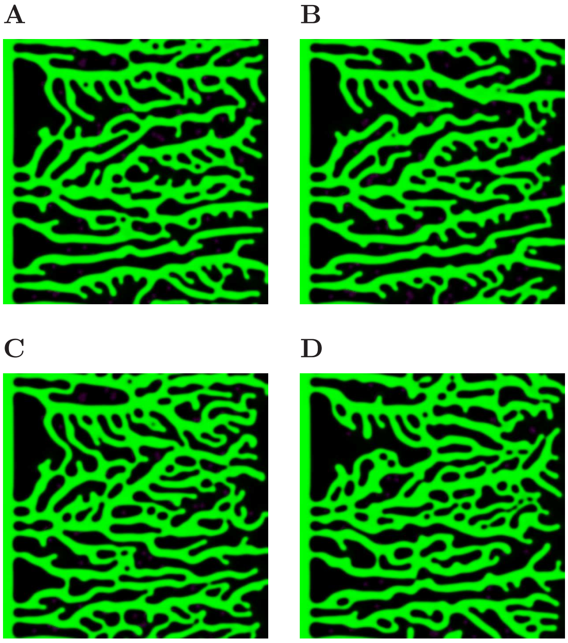

Figure 4
